# Dietary Macronutrient and Micronutrient Intake over a 7-Day Period in Female Varsity Ice Hockey Players

**DOI:** 10.3390/nu13072262

**Published:** 2021-06-30

**Authors:** Tyler F. Vermeulen, Logan A. Boyd, Lawrence L. Spriet

**Affiliations:** Department of Human Health and Nutritional Sciences, University of Guelph, Guelph, ON N1G 2W1, Canada; loganboyd105@gmail.com (L.A.B.); lspriet@uoguelph.ca (L.L.S.)

**Keywords:** nutrition, female athletes, ice hockey, macronutrients, micronutrients, energy intake, fluid intake

## Abstract

This study examined the energy, macronutrient, and micronutrient intakes of female ice hockey players over a 7-d period including game, practice, and rest days. Twenty-three female varsity players (19.0 ± 1.1 yr, 167.1 ± 6.5 cm, 67.0 ± 8.0 kg) volunteered for the study. Average total daily energy expenditure (TDEE) was estimated over the 7-day period. Average 7-day energy intake (EI) and TDEE were 2354 ± 353 and 2304 ± 204 kcal. The majority (n = 19) of athletes had an EI ≥ 90% of their estimated TDEE. Macronutrient intake was 52% carbohydrate (CHO), 32% fat, and 16% protein of total EI, although CHO intake was slightly below recommendations (5 g/kg BM/d) on game and practice (4.8 ± 1.4 and 4.5 g/kg BM/d) days. Game day EI was greater than practice and rest days. Recommended micronutrient intakes were not met by most athletes for iron, calcium, vitamin D, and potassium, and intakes were similar between game, practice, and rest days. In summary, the average EI for female varsity ice hockey players appeared adequate to meet their energy needs over a weekly cycle of game, practice, and rest days. However, these female athletes would benefit from increasing CHO intake on game and practice days and selecting foods that are rich in vitamins and minerals.

## 1. Introduction

Ice hockey is a stop-and-go team sport that can be characterized by repeated bouts of high-intensity activity combined with periods of active low-intensity recovery or passive rest [1,2]. To meet the energy demands and nutritional needs of an intense training regimen, an athlete needs a well-chosen diet that provides the energy necessary for peak physiological functioning and includes a wide variety of foods rich in vitamins and minerals. Elite ice hockey players require exceptional speed, power, endurance, and explosiveness, which is fueled by both the anaerobic and aerobic energy systems [3,4,5]. Adequate energy intake (EI) and carbohydrate (CHO) consumption is necessary to fuel these systems. However, a recent review concluded that team sport athletes often do not meet their dietary intake recommendations [6] and many studies with female athletes in stop-and-go sports such as soccer and basketball have also reported insufficient EI [7,8].

Research has shown that inadequate EI (as compared to total daily energy expenditure (TDEE) can negatively affect performance and lead to adverse health outcomes such as loss of muscle mass, increased susceptibility to injuries, and immune and endocrine disturbances [9]. In addition to meeting EI needs, the foods consumed must provide the micronutrients that are essential for many physiological processes, including energy synthesis, metabolic control, and hormone production [9,10]. Micronutrients that female athletes commonly consume in low quantities include iron, calcium, and vitamin D [11]. In a study of female elite soccer players, it was reported that despite energy needs being met through dietary intake, the minimum recommended intake of multiple micronutrients was not met [12]. In addition, several studies have shown that female athletes are at an increased risk for macronutrient and micronutrient deficiencies [13,14,15]. 

In summary, while proper nutrition is a key component to health and performance, female athletes are at an increased risk of nutrient deficiencies, eating disorders, and dietary-related health issues compared to their male counterparts [16]. Due to the high demands of training and a sport culture often centered around aesthetics, female athletes often report low EI from either under-consumption and/or under-reporting [17]. At present, we are unaware of any dietary research involving female ice hockey players, as the available research has examined only male athletes [18,19]. Therefore, the primary aim of this study was to examine the energy, macronutrient and micronutrient dietary intake of university level female ice hockey players over a 7-day training phase that included game, practice, and rest days. As a secondary aim, we also compared the average weekly EI of players to an estimation of their average weekly energy expenditure. We hypothesized that this group of athletes would meet, or be close to, their energy and macronutrient recommendations when undertaking a carefully controlled 7-day dietary record.

## 2. Materials and Methods

### 2.1. Participants

Twenty-three female varsity ice hockey players (19.0 ± 1.1 yr, 167.1 ± 6.5 cm, 67.0 ± 8.0 kg) from a University women’s hockey team participated in this study. All participants were familiarized with ice hockey and had experience playing since their early childhood. All players from the varsity team were invited to participate and all volunteered (n = 27). The only exclusion criterion was if the player did not play games during the week that their 7-day diet record was collected (n = 4). Each participant provided oral and written informed consent prior to the study. The study was approved by the Research Ethics Board of the University of Guelph (REB#16JL010), Canada. 

### 2.2. Experimental Overview

Nutritional intake was assessed using a 7-day dietary record. Collection of dietary data occurred over 7 consecutive days (a typical training and competition week) during October and November of the competitive season and provided information on the type and quantity of foods and fluids consumed daily. Dietary records were analyzed for energy and micronutrient intake. Data were collected from 4–6 athletes each week to allow for daily individual meetings with the researcher. Each 7-day period (Monday to Sunday) included ~1 home game, 1 away game, 3 practice days, and 2 rest days. Practices included on- and off-ice training. On-ice sessions included skating and stickhandling drills, skills development, and conditioning that lasted for 60–90 min/practice. Longer and more rigorous practices occurred at the start of the week with a tapering into less-rigorous practices that focused on skill development from the mid to end of week. Off-ice training did not typically coincide with on-ice sessions; however, several days did include both on- and off-ice training. The off-ice sessions were a combination of strength/resistance, cardiovascular, and high-intensity interval training (HIIT) and lasted ~45–60 min. Supplement use was recorded but excluded from the dietary intake data. 

### 2.3. Measurements

Participant age (yr) was reported verbally and standing height (cm) was measured using a wall-mounted measuring scale. Body mass (BM) was measured in kilograms (kg) using a digital scale accurate to ±0.1 kg (Zenith, LG Electronics Canada, Mississauga, ON, Canada). Anthropometric measures were recorded prior to beginning the dietary record. 

TDEE was estimated based on the athlete’s predicted basal metabolic rate (BMR), estimated exercise energy expenditure (EEE), and the thermic effect of food (TEF) using the equation as described by Nelms et al. [20]: TDEE = BMR + EEE + TEF. BMR was estimated by multiplying resting oxygen intake (3.5 mL O_2_/min) by body mass (BM) in kg. This product was then multiplied by min in an hour (min/h) along with hours at rest during a day (h/day), divided by 1000 (to convert mL to Litres) and multiplied by 4.85 kcal/L O_2_ (assuming 50/50 use of fat and carbohydrate). The TEF was calculated by multiplying total EI by 10%. Lastly, the EEE was calculated by multiplying 0.0175 kcals (a constant) by the estimated metabolic equivalents for women playing ice hockey (7.3) and BM (kg).

### 2.4. Recording of Nutritional Intake

Several measures were employed to maximize the accuracy of the athlete’s dietary records as recording energy intake can be problematic [16,21,22]. Athletes that cohabitated (4–6) were studied during a given week and each athlete was provided with a food scale (Starfrit electronic kitchen scale, Longueuil, QC, Canada), recording sheets, reference charts, and food pictures. Athletes were given verbal and written instructions on how to use the food scale and record daily food and fluid intake prior to beginning the study. For 7 consecutive days, athletes recorded information about all food and fluids consumed (e.g., type, brand, and amount). Athletes were also instructed to record any dietary supplements consumed over the 7-day period. To provide a realistic representation of a typical diet within this population, athletes were encouraged to eat as they normally would and to be honest about all foods consumed. When the use of a food scale was not possible while dining out, instructions were given to take photos of the food before and after eating, from a top and side view, to determine the composition of food and amount consumed. Daily meetings were also conducted with every athlete to check the records for completion and accuracy. Food records were analyzed using ESHA nutritional software (ESHA Processor Nutrition Analysis Software, Salem, OR, USA). Energy and macronutrient intakes were compared between game, practice, and rest days, and against the American College of Sports Medicine Joint Position Statement [23]. Micronutrient intake was compared between game, practice, and rest days, and against recommendations from Canada’s Dietary Reference Intake (DRI) values [24]. Intakes of calcium, iron, Vitamin D, and sodium were investigated. 

### 2.5. Statistical Analyses

Data were analyzed using IBM SPSS Statistics, Version 26 (IBM Corp., Armonk, NY, USA). Descriptive statistics were presented as mean + SD. The Shapiro–Wilk test was used to identify non-normal data. A two-tailed paired t-test was used to compare EI and TDEE. A one-way analysis of variance was used to compare macronutrient and micronutrient data between game, practice, and rest days. Post hoc analyses were performed with Tukey’s multiple comparison tests when a significant F ratio was found. Statistical significance was *p* ≤ 0.05.

## 3. Results

**Figure 1:**
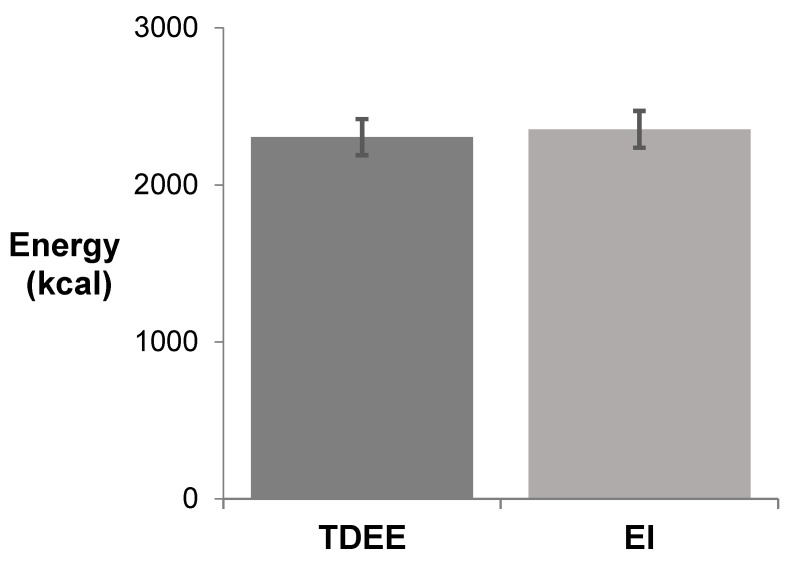
Means ± SD, n = 23. Total daily energy expenditure (TDEE) compared to energy intake (EI) for team over a 7-day period of game, practice, and rest days.

The average EI over 7 days was 2354 ± 355 kcal and not significantly different from the estimated average TDEE of 2304 ± 204 kcal (Figure 1). The majority of athletes had an EI that reasonably matched their TDEE, with only 17% (n = 4) athletes having an EI >10% lower than TDEE estimates (79, 81, 87, and 89%). Conversely, 30% (n = 7) athletes had an EI >10% higher than TDEE estimates (111, 114, 115, 115, 116, 124, and 126%).

The absolute amounts and proportions of each macronutrient consumed/day over the 7-day period was 305 g and 52% from CHO, 82 g and 32% from fat, and 91 g and 16% from protein (Table 1). Fluid intake averaged 2162 mL/day.

The average training load over the 7-day period included 2 games, 3 practices, and 2 rest days. EI was higher on game days than practice (*p* < 0.05) and rest (*p* < 0.002) days (Table 2). EI expressed per kg BM was also higher on game vs. rest (*p* < 0.002) days, but not practice days. Carbohydrate intake was not different between days, but fat intake was higher on game vs. rest days when expressed in grams (*p* < 0.03) and per kg BM (*p* < 0.04). Protein intake was also greater on game vs. rest days when expressed in grams (*p* < 0.009) and per kg BM (*p* < 0.009), and practice day protein intake was higher than rest when expressed per kg BM (*p* < 0.05). Fluid intake in mL on game days was greater than practice (*p* < 0.03) and rest days (*p* < 0.001), and also greater when expressed per kg BM (*p* < 0.03 and *p* < 0.02, respectively) (Table 2).

**Figure 2:**
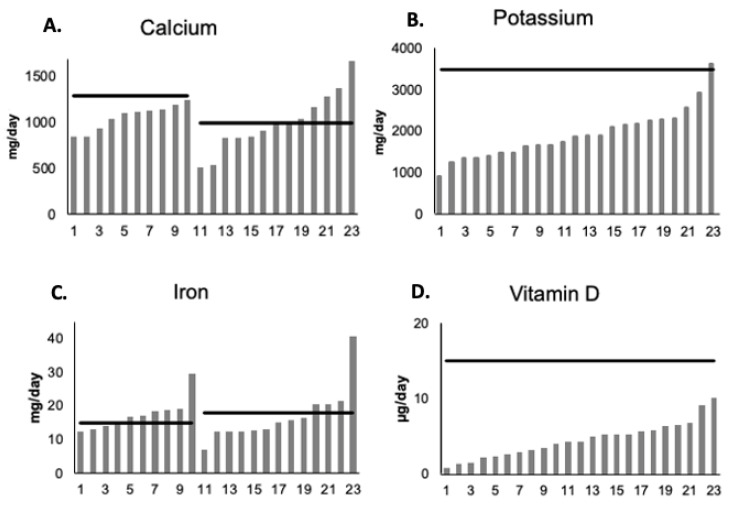
Average daily intake of (**A**). calcium (RDA = 1000–1300 mg), (**B**). potassium (recommended dietary allowance (RDA = 3500 mg), (**C**). iron (RDA = 15–18 mg), and (**D**). vitamin D (RDA = 15 μg). n = 23. Participant data listed from lowest to highest intake.

The majority of athletes met the daily recommendations for sodium, manganese, selenium, vitamin B_12_ and vitamin C intakes when compared to Canadian DRI recommendations (Table 3). However, less than half (48%) of the athletes met recommendations for iron, only 22% (n = 5) met the calcium recommendations, and no athletes met the recommendations for vitamin D intake (Table 3, Figure 2). In addition, several athletes did not meet the recommendations for magnesium, vitamin E, potassium, phosphorus, copper, folate, and zinc (Table 3). 

There were no differences in micronutrient intakes between game, practice, and rest days (Table 4).

## 4. Discussion

To our knowledge, this is the first study to measure the nutritional intake of elite female ice hockey players using a carefully measured 7-day dietary record. The key findings from this study were as follows: (1) in general, athletes had an energy intake (EI) that appeared sufficient to match their estimated total daily energy expenditure (TDEE) thereby supporting our hypothesis; (2) macronutrient intake was well proportioned at 52% carbohydrate, 32% fat and 16% protein of total energy intake, although CHO intake was slightly below recommendations on game and practice days; (3) athletes had higher EIs on game vs. practice and rest days based on greater fat and protein consumption; (4) many athletes did not meet the recommended daily intakes (RDI) of key micronutrients, including iron, calcium, vitamins D, and potassium, and (5) no differences in micronutrient intake were found between game, practice or rest days. 

### 4.1. Energy Expenditure vs. Energy Intake

Generally, the EI of these female ice hockey players matched their estimated TDEE when averaged over a 7-day period. This finding is in contrast to previous findings in Canadian high-performance athletes [25] and several studies of female team sport athletes that reported a negative energy balance [8,26,27,28]. However, the authors of these studies suggested that the energy deficit may have been, in part, due to the underreporting of dietary intakes. We also realize that we did not employ “gold standard” techniques to measure energy expenditure in the present study. In addition, our data showed that the players also did well in meeting their macronutrient recommendations, with CHO being the only macronutrient slightly below recommendations. However, more CHO-based foods were consumed on game days over practice and rest days and resulted in CHO periodization. This higher CHO intake is needed for game days as a high-intensity stop-and-go sport like ice hockey requires CHO for its dominant fuel, especially for the high exertions seen during a game. 

However, unlike the previously mentioned studies that used 3- to 4-day dietary records, this study used 7-day dietary records to include both weekdays and weekends, as well as game, practice, and rest days to encompass a typical week for these athletes. Additionally, to help mitigate underreporting, food scales were used to accurately record all food and beverages, and before and after pictures were used when food scales could not be used while dining out. Further, athletes were studied in groups that shared similar living locations to increase compliance and daily meetings were conducted with each athlete to ensure any inconsistencies in reporting were minimized. We believe that the compliance of the athletes to accurately record their dietary intake over their weekly training and competing cycle may explain the relatively strong match between EI and TDEE. Even if the present EI and TDEE measures were in error by 10% above or below the current values, the conclusion that these athletes were generally consuming enough food to meet their daily energy demands would hold.

### 4.2. Macronutrient Intake

The absolute amounts of CHO and protein intakes over the 7-day dietary record were 4.6 ± 1.0 and 1.4 ± 0.5 g/kg BM/day, respectively. As a team, the absolute CHO intake was below the recommendation of 5–7 g/kg BM/day, while protein intakes met the recommendation of 1.2–2.0 g/kg BM/day [23]. Although the players were below the absolute CHO recommendation of 5–7 g/kg BM/day, they did consume more CHO on game days compared to practices and rest days. These greater intakes on game days resulted in a CHO periodization, where the athlete ate more CHO on the days of heavier exertions, such as a competitive game, to meet fueling demands. Less CHO on practice and rest days was acceptable. The recommendation for athletes to consume 20–35% of their total daily energy as fat was also closely met (32% fat intake/day). 

There were significantly greater intakes of total energy, fat, and protein between game and rest days and in fluid intake on game vs. rest and practice days. The greater energy intake from fat and protein on game days was attributed to higher consumption of eggs, cooking oils, nuts, and sport supplement protein bars. There was a small increase in CHO intake on game days (319 g) vs. practice (301 g) and rest (297 g) days, but this increase was not significant. 

The majority of CHO utilized during high-intensity training comes from the breakdown of muscle glycogen [29,30]. With CHO being used as a fuel for both anaerobic and aerobic conditions, it is relied heavily upon during stop-and-go sports like ice hockey [31]. The suggested minimum CHO requirement to maintain glycogen stores and support general training of all athletes in various training situations is ~5 g CHO/kg BM/day [32]. However, ranges from ~5 to 7 g/kg BM/day are recommended for moderate exercise programs while 6–10 g/kg BM/day are listed for higher endurance programs [23]. As a team, the players were slightly below the 5 g/kg BM/day in the present study, with an intake of 4.6 ± 1.0 g/kg BM/day for the entire 7 days and intakes of 4.8 and 4.5 g/kg BM/day for game and practice days. These finding were higher than reported in female field hockey players where amounts of 3.4–4.3 g/kg BM/day were consumed [33,34,35]. Higher CHO intakes amongst female team sports athletes were reported in collegiate basketball (5.3 g/kg BM/day) and soccer (5.2 g/kg BM/day) players [12,36]. In conclusion, the CHO intake in the present study revealed that these ice hockey athletes were near the suggested recommendations of ~5 g CHO/kg BM/day but may benefit from consuming more CHO on practice and game days. 

### 4.3. Micronutrient Intake

Despite the majority of athletes in the current study having an EI ≥ 90% of their TDEE, many athletes did not meet the recommendations for micronutrient intakes based on the average of the 7-day period. The micronutrient intake between game, practice, and rest days indicated no differences between types of days. The findings of this study were similar to that reported for NCAA female soccer players who failed to meet recommendations for micronutrient intakes despite having an EI that met their caloric needs [12]. A possible explanation for these findings may be that processed foods high in calories displaced nutrient-dense foods in their diets.

Currently, there is no clear consensus on whether micronutrient needs differ in athletes compared to the general population, and athletes are often advised to meet general recommendations through a diverse diet [37]. Due to the increased stress that exercise puts on metabolic pathways that require micronutrients, it is important for athletes to at least meet recommendations for the general population. Micronutrients of particular concern in athletes that play a role in performance, recovery, and health include vitamin D, iron, and calcium [23]. In the current study, all three of these micronutrients were under consumed by over half of the athletes. Additionally, a large majority of athletes also reported inadequate intake of potassium, magnesium, and vitamin E. Conversely, most athletes met recommendations for manganese, selenium, vitamin B_12_, and vitamin C intake, which suggests these micronutrients may be more easily consumed through food.

Of the few studies that have reported vitamin D intake in female athletes, intake has consistently been far below the recommended intake of 15 μg/day [38,39]. Similar results were seen in this study, with an average intake of 4.5 ± 2.4 μg/day and none of the athletes meeting the recommended intake through food alone. Because of the important role vitamin D plays in the maintenance of bone health and in the regulation of calcium and phosphorus absorption, supplementation may be necessary. It has been shown that male and female athletes that train indoors during the fall and winter months suffer from low vitamin D levels [40]. Since vitamin D is only found in limited natural and fortified foods such as fatty fish, mushrooms, egg yolks, and cereals, it is unlikely that adequate vitamin D status can be maintained through food alone in the present athlete population [41].

Iron is also a micronutrient of particular concern for female athletes, often due to restricted EI, lack of consumption of iron-rich foods, and increased iron losses [42,43]. Since the majority of athletes in this study had adequate EI, it suggests that about 50% of the players who did not meet the RDI may not be consuming enough iron rich foods with a high iron bioavailability such as meat, fish, and poultry. 

Calcium is associated with the growth, maintenance, and repair of bone tissue, along with the regulation of muscle contraction and nerve impulse transmission [23]. Calcium recommendations are 1000 mg/day which the team met with an average intake of 1022 ± 256 mg/day. Calcium intake was higher on practice and game days at ~1100 mg vs. rest days (930 mg) although not significantly. Of note was that only five players met the calcium intake recommendations over the 7-day period. Calcium levels can be vastly improved via dairy consumption, assuming that lactose intolerance is a non-issue.

### 4.4. Fluid, Sodium, and Potassium Intakes

Absolute fluid intakes were below recommendations (~2700 mL/day) on practice and rest days but were above 2700 mL on game days. Sodium is a micronutrient that may be required in greater amounts for female athletes compared to their sedentary counterparts, as they train and compete in hot macro/microenvironments and lose sodium through high sweating rates [44]. Replacing the sodium lost through sweat is important during and after exercise in order to maintain fluid balance within the body [45]. In this study, all but one athlete had an average sodium intake above the upper limit of 2300 mg/day, indicating that sodium balance was likely not a risk despite increased losses through sweat. 

An interesting finding of this study was that athletes were consuming far less potassium than the recommended 3500 mg/day with a team average of 1921 ± 596 mg/day. Ziegler et al. [15] reported a similar potassium intake among female figure skaters, but Condo et al. [46] reported that over half of female Australian rules football players had adequate potassium intake, however the RDI used for comparison was 2800 mg/day. Discrepancies in RDI values and a consistently inadequate intake in female athletes makes it difficult to determine if potassium needs are being met. More studies are needed to determine a definitive potassium intake.

### 4.5. Limitations

Although this study provides valuable insight into the dietary intake of female varsity ice-hockey players, there are limitations that must be acknowledged. First, although the 7-day dietary record may provide a more accurate representation of dietary intake compared to shorter assessments of dietary intake and dietary recall methods, it still is dependent on self-reporting. Self-reporting relies largely on honesty and compliance and previous studies with female athletes have documented under-reporting of dietary intake when self-reporting [21,47,48]. However, daily meetings with athletes and the use of food scales may have helped to mitigate any effects of self-reporting on data accuracy. We also recognize that there are many assumptions in the prediction of average daily energy expenditure that was used in the present study and more accurate measures such as activity monitors or doubly-labelled water techniques are needed to improve the accuracy of these estimates. 

## 5. Conclusions

In summary, female varsity ice hockey players appeared to consume adequate energy for their daily energy requirements over a 7-day cycle of games, practices, and rest days. Players consumed more energy on game vs. practice and rest days and relative percent macronutrient intakes were 52% carbohydrate, 32% fat and 16% protein. Absolute carbohydrate intake was ~4.5–4.8 g/kg BM on all days and slightly below the recommendation of ~5 g/kg BM/day. Protein intake was 1.2–1.5 g/kg BM and within the recommended intake for athletes. Many athletes did not meet general recommendations for several micronutrients through dietary intake alone (i.e., vitamin D, iron, calcium, and potassium). These deficiencies may be the result of poor dietary choices and a high consumption of refined foods. These athletes would benefit from nutritional education so they have a better understanding how to meet their micro- and macronutrient needs and how fueling their bodies can impact their performance. Therefore, these female varsity ice hockey athletes would benefit from (1) working with a sports dietitian or nutritionist to increase their nutritional knowledge and understand how to fuel themselves properly during competition, training, and recovery days, (2) increasing their CHO intake on game and practice days, and (3) consuming a diet richer in vitamins and minerals. 

## Figures and Tables

**Table 1:** Energy, macronutrient, and fluid intakes of varsity female ice hockey players.

Dietary Measure	Recommendation	Average	Range	Athletes Meeting Recommendations % (n)
Energy (kcal)		2354 ± 355	1533–3029	
(kcal/kg)		35 ± 5	27–47	
Carbohydrate (% energy)		52 ± 5	36–65	
(g)		305 ± 72	178–476	
(g/kg)	5–7 g/kg	4.6 ± 1.0	2.5–7.6	39% (n = 9)
Fat (% energy)		32 ± 5	21–42	
(g)		82 ± 19	49–117	
(g/kg)	<30%; with no more than 10% from saturated fats	1.2 ± 0.5	0.7–1.7	35% (n = 8)
Protein (% energy)		16 ± 5	10–21	
(g)		91 ± 19	56–148	
(g/kg)	1.2–2.0 g/kg	1.4 ± 0.5	1.0–1.8	87% (n = 20)
Fluid (mL)		2162 ± 758	1158–3453	
(mL/kg)		32.1 ± 10.0	17.6–52.0	

Means ± SD, n = 23. Based on averaged 7-day dietary intakes. Recommendations from Thomas et al. [23].

**Table 2:** Energy, macronutrient, and fluid intakes for game, practice, and rest days for varsity female ice hockey players.

Dietary Measure	Games	Practices	Rest
Energy (kcal)	2616 ± 508 **	2317 ± 436	2146 ± 441
(kcal/kg)	39.3 ± 7.7 *	34.9 ± 7.2	32.2 ± 6.2
Carbohydrate (% energy)	48 ± 10	52 ± 10	55 ± 10
(g)	319 ± 91	301 ± 77	297 ± 82
(g/kg)	4.8 ± 1.4	4.5 ± 1.4	4.5 ± 1.0
Fat (% energy)	30 ± 5	33 ± 7	31 ± 10
(g)	87 ± 24 *	85 ± 22	73 ± 24
(g/kg)	1.3 ± 0.5 *	1.3 ± 0.5	1.1 ± 0.5
Protein (% energy)	15 ± 5	16 ± 5	15 ± 5
(g)	97 ± 29 *	93 ± 19	82 ± 24
(g/kg)	1.5 ± 0.5 *	1.4 ± 0.5 *	1.2 ± 0.5
Fluid (mL)	2739 ± 930 **	2071 ± 820	1721 ± 777
(mL/kg)	40.5 ± 11.0 **	30.7 ± 11.0	25.7 ± 11.0

Means ± SD, n = 23. The average training load over the 7-day period included 2 games, 3 practices, and 2 rest days. *, significantly different from rest days. **, significantly different from practice and rest days.

**Table 3:** Average 7-day micronutrient intakes for female ice hockey players, and percent of athletes meeting recommended micronutrient intakes.

Dietary Measure	Recommendation	Average	Athletes Meeting Recommendations % (n)
Calcium (mg)	1000–1300 mg/day	1022 ± 256	22% (n = 5)
Vitamin D (μg)	15 μg/day	4.5 ± 2.4	0% (n = 0)
Iron (mg)	15–18 mg/day	17 ± 7	48% (n = 11)
Sodium (mg) ^1^	1500 mg/day	3266 ± 667	100% (n = 23)
Magnesium (mg)	310–360 mg/day	219 ± 6	9% (n = 2)
Zinc (mg)	8–9 mg/day	7.7 ± 2.2	39% (n = 9)
Potassium (mg)	3500 mg/day	1921 ± 596	4% (n = 1)
Phosphorus (mg)	700–1250 mg/day	947 ± 261	43% (n = 10)
Copper (mg)	0.89–0.9 mg/day	0.9 ± 0.3	48% (n = 11)
Manganese (mg) ^1^	1.6–1.8 mg/day	2.5 ± 1.0	83% (n = 19)
Folate (μg) (DFE)	400 μg/day	413 ± 240	43% (n = 10)
Vitamin E (mg)	15 mg/day	7.6 ± 3.7	9% (n = 2)
Selenium (μg)	55 μg/day	98 ± 30	91% (n = 21)
Vitamin B_12_ (μg)	2.4 μg/day	3.9 ± 2.2	70% (n = 16)
Vitamin C (mg)	65–75 mg/day	109 ± 40	83% (n = 19)

Means ± SD, n = 23. Recommendations are based on Health Canada Dietary Reference Intakes. ^1^ Recommendations based on adequate intake. Remaining recommendations based on recommended dietary allowance. DFE = dietary folate equivalent.

**Table 4:** Selected micronutrient intakes for game, practice, and rest days in varsity female ice hockey players.

Dietary Measure	Games	Practices	Rest
Calcium (mg)	1108 ± 508	1104 ± 470	931 ± 466
	(178–2422)	(189–2770)	(296–1841)
Vitamin D (μg)	5.2 ± 5.3	4.0 ± 3.1	4.5 ± 3.7
	(0.0–24.6)	(0.0–13.5)	(0.5–13.0)
Iron (mg)	18 ± 13	17 ± 9	17 ± 9
	(4–80)	(3–48)	(6–38)
Sodium (mg)	3283 ± 1210	3333 ± 1730	3330 ± 1109
	(706–7248)	(960–13925)	(1523–6013)
Magnesium (mg)	245 ± 116	209 ± 97	210 ± 103
	(59–678)	(32–494)	(53–401)
Zinc (mg)	7.8 ± 3.8	7.7 ± 4.3	7.6 ± 4.5
	(1.0–18.6)	(0.4–23.3)	(0.8–19.6)
Potassium (mg)	2166 ± 993	1840 ± 862	1750 ± 785
	(561–4843)	(266–4527)	(466–2868)
Phosphorus (mg)	1046 ± 499	903 ± 409	926 ± 436
	(167–2618)	(44–2139)	(104–1665)
Copper (mg)	1.1 ± 0.5	0.9 ± 0.4	0.9 ± 0.5
	(0.3–2.5)	(0.2–2.0)	(0.2–2.1)
Manganese (mg)	2.8 ± 2.0	2.3 ± 1.8	2.4 ± 1.8
	(0.7–11.7)	(0.1–13.5)	(0.5–7.7)
Folate (μg DFE)	416 ± 396	412 ± 342	413 ± 289
	(53–2261)	(10–1358)	(57–1161)
Vitamin E (mg)	7.9 ± 9.9	8.1 ± 8.3	5.0 ± 3.8
	(0.6–55.9)	(0.2–39.0)	(1.2–16.5)
Selenium (μg)	107 ± 60	92 ± 52	99 ± 47
	(11–249)	(1–270)	(2–191)
Vitamin B_12_ (μg)	3.9 ± 3.5	4.0 ± 3.9	3.5 ± 2.7
	(0.0–15.2)	(0.0–25.8)	(0.1–8.6)
Vitamin C (mg)	114 ± 71	114 ± 85	84 ± 76
	(0–347)	(0–389)	(0–257)

Means ± SD, (range), n = 23. Data based on averages from 2 games, 3 practices, and 2 rest days. DFE = dietary folate equivalent.
